# Resolution of acute rejection in a bilateral double-lung transplanted cystic fibrosis patient following phage intervention

**DOI:** 10.1128/asmcr.00058-24

**Published:** 2025-07-23

**Authors:** Marco Pardo-Freire, Mireia Bernabéu-Gimeno, Benjamin K. Chan, Paul E. Turner, Isabel Sánchez-Romero, Myriam Aguilar-Pérez, Marta Erro-Iribarren, Pilar Domingo-Calap

**Affiliations:** 1 Institute for Integrative Systems Biology, University of Valencia-CSIC 588140, Paterna, Spain; 2 Department of Ecology and Evolutionary Biology, Yale University 5755, New Haven, Connecticut, USA; 3 Center for Phage Biology and Therapy, Yale University 5755, New Haven, Connecticut, USA; 4 Program in Microbiology, Yale School of Medicine 12228 , New Haven, Connecticut, USA; 5 Department of Microbiology, Hospital Universitario Puerta de Hierro, Madrid, Spain; 6 Department of Pneumonology, Hospital Universitario Puerta de Hierro, Madrid, Spain; Pattern Bioscience, Austin, Texas, USA

**Keywords:** cystic fibrosis, phage therapy, antimicrobial resistance, lung transplantation

## Abstract

**Background:**

Antimicrobial resistance is a major threat to human health worldwide, requiring investigation into alternative therapeutics such as phage therapy. Phages have been used to treat numerous multidrug-resistant infections recently, particularly in people with cystic fibrosis (CF) who are predisposed to infection from *Pseudomonas aeruginosa*.

**Case Summary:**

Here, we report the use of phage therapy to treat a chronic *P. aeruginosa* infection in a 44-year-old woman with CF who underwent bilateral lung transplantation. This individual experienced acute rejection and deteriorating lung function, possibly worsened as a consequence of a *P. aeruginosa* infection. Phage therapy using phage vB_Pae_10 was administered via nebulizer alongside standard antimicrobials in two 10-day courses. Clinical and microbiological assessments indicated an initial reduction in bacterial load along with an improvement in lung function. Phage therapy was well-tolerated with no adverse events reported. The patient showed sustained clinical improvement, including mucus clearance, enhanced pulmonary function, and resolution of acute rejection.

**Conclusion:**

This case underscores the potential of phage therapy to complement traditional treatments in managing chronic bacterial infections in CF patients, though further studies are needed to optimize treatment protocols and understand the role of phages in modulating the bacterial population.

## INTRODUCTION

Antibiotic resistance is a major threat to cystic fibrosis (CF) patients, whose impaired mucus clearance facilitates lung bacterial infection (1). Long-term antibiotic treatments select for multidrug-resistant (MDR) strains (2). These infections promote inflammation, tissue damage, and lung function loss, key factors in CF morbidity and mortality (3). Lung transplantation is an option in case of advanced damage but does not prevent recolonization or new infections (4). Limited treatments for MDR bacteria increased the interest in bacteriophages or phages (viruses that infect bacteria) (5–7). The absence of a regulatory framework in most countries restricts phage therapy to compassionate use, with variable dosages, durations, and administration routes, making it difficult to draw conclusions and design clinical trials. We describe a phage treatment in a bilateral double lung transplantation CF patient with a *Pseudomonas aeruginosa* lung infection potentially complicating the acute rejection. Two 10-day phage nebulization courses combined with antibiotics led to an initial reduction of the bacterial load and long-term improvement of the lung function. The patient’s clinical status improved, ultimately leading to the resolution of acute rejection.

## CASE PRESENTATION

**Fig 1:**
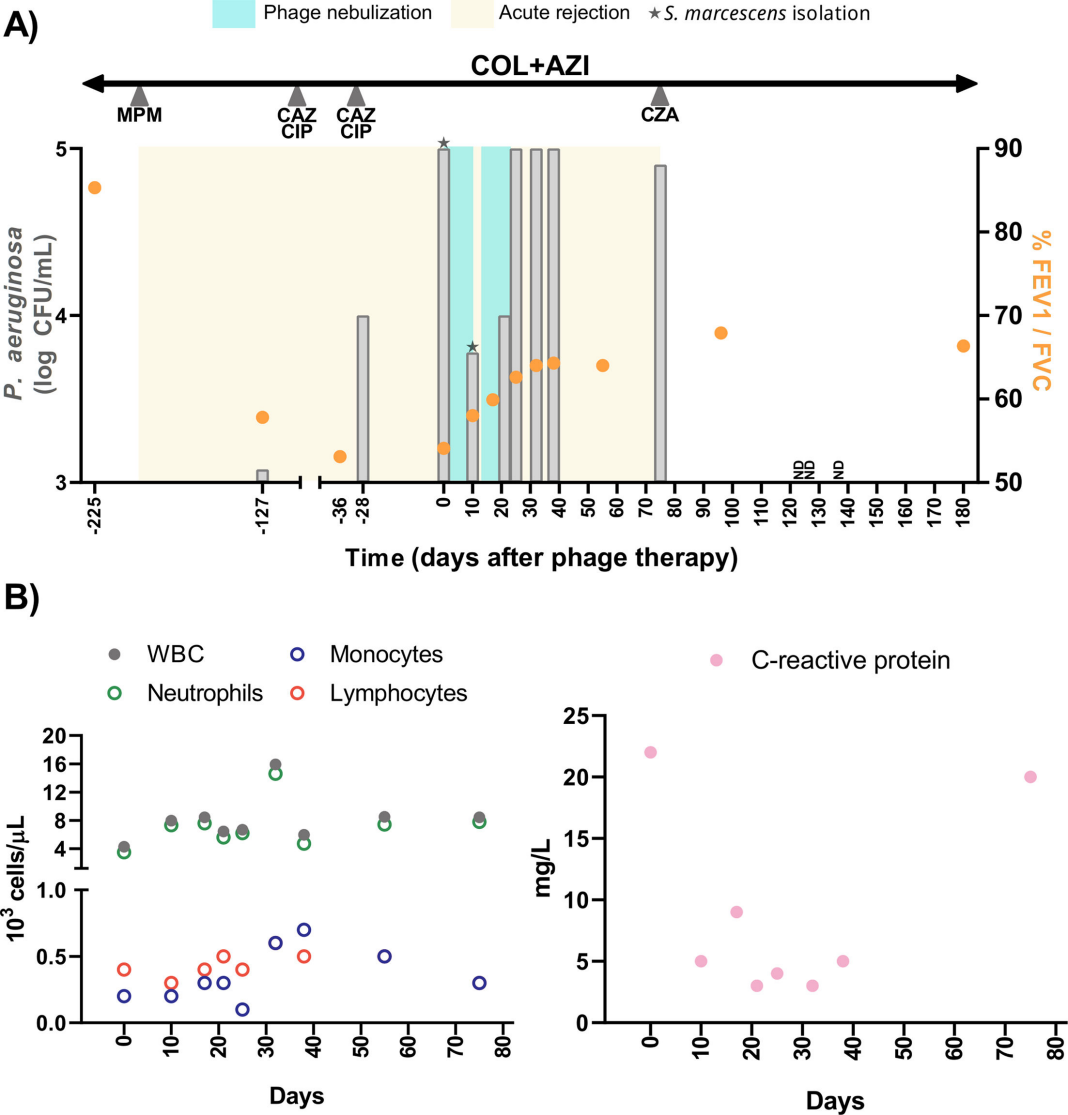
Microbiological and clinical outcome of phage treatment. (**A**) Timeline including *P. aeruginosa* load in sputum samples (left *y*-axis, in gray bars) and %FEV1/FVC ratio by spirometry (right *y*-axis, in orange). Sputum samples were mixed with N-acetylcysteine prior to bacterial isolation. Duration of the acute rejection status is represented in yellow, two rounds of 10-day phage treatment in blue, and antibiotics taken during this period are included at the top of the graph with the start of intravenous treatments marked with a gray arrow. Samples from which *Serratia marcescens* could be isolated are marked with a star. ND: no detection of bacteria in sputum. MPM: meropenem. COL: colistin. AZI: azithromycin. CAZ: ceftazidime. CIP: ciprofloxacin. CZA: ceftazidime-avibactam. (**B**) White blood cell count (left) and C-reactive protein (CRP) concentration (right) measured since treatment (day 0). The left graph shows the total white blood cell count (WBC) along with individual values for neutrophils, monocytes, and lymphocyte counts as 10^3^ cells/µL. The right graph shows CRP measured as mg/L.

The patient was a 44-year-old woman with CF (homozygous for ΔF508) who underwent bilateral pulmonary transplants at the ages of 29 and 40 years. She had a chronic *P. aeruginosa* lung infection since childhood, with intermittent colonization by *Aspergillus* spp., *Serratia marcescens,* and *Stenotrophomonas maltophilia*. Following the latest transplant, her condition remained stable (no clinical symptoms related to bacterial infections) under chronic medication (Table 1). In May 2023, her pulmonary function, measured as the ratio of forced expiratory volume in one second (FEV1) to forced vital capacity (FVC), was 85% (Fig. 1). In June, a transbronchial biopsy revealed acute cellular rejection grade A2B2, which required additional immunosuppressive treatments (Table 1). By the end of August, %FEV1/FVC declined to 57.8%, and *P. aeruginosa* was isolated from a bronchoalveolar lavage (BAL) sample, prompting the first intravenous (IV) ceftazidime and ciprofloxacin treatment. The persistent rejection worsened her lung function and overall condition, and in October, she received multiple ineffective IV antibiotic cycles for the *P. aeruginosa* infection (Table 1), suspected to worsen the acute rejection. By November 2023, %FEV1/FVC decreased to its lowest (53.1%), while bacterial load in BAL continued increasing (measured by colony-forming units (CFU)/mL using quantitative seeding (8), and a second IV ceftazidime and ciprofloxacin treatment was prescribed. As *in vitro* antimicrobial susceptibility testing in isolates from CF patients is unreliable (9), the drug regimen was based on tolerability and clinical response. Given the potential implications of the *P. aeruginosa* infection in exacerbating the graft rejection and the ineffectiveness of conventional antibiotics, phage therapy was proposed.

**TABLE 1:** Patient’s prescriptions before, during, and after phage therapy^*a*^

Category	Treatment	Administration	Time period	Concomitant use with phage
Immunosuppressor	Tacrolimus	1 mg, orally, every 12 h	Chronic	Yes
	Everolimus	1 mg, orally, every 12 h	Chronic	Yes
	Prednisone	10 mg, orally, every 24 h	Chronic	Yes
	Corticosteroid	Bolus	June, July, and August 2023	No
	Thymoglobulin	Bolus	Oct-23	No
	Photopheresis	NA	Since November 2023	Yes
Antibacterial	Azithromycin	500 mg, orally, three times per week	Chronic	Yes
	Colistin	2 MIU, nebulized, every 8 h	Chronic	Yes
	Meropenem	Intravenously	June and October 2023	No
	Ceftazidime + ciprofloxacin	Intravenously	September and December 2023	No
	Cotrimoxazole	Intravenously	Oct-23	No
	Ceftozolane/tazobactam	Intravenously	Oct-23	No
	Piperacillin/tazobactam	Intravenously	Oct-23	No
	Ceftazidime/avibactam	Intravenously	Mar-24	No
Antiviral	Valganciclovir	900 mg, orally, every 24 h	Chronic	Yes
Antifungal	Voriconazole	40 mg, nebulized, every 12 h	Chronic	Yes
	Posaconazole	300 mg, orally, every 24 h	Chronic	Yes
CFTR modulator	Elexacaftor/tezacaftor/ivacaftor	NA	From day 67 to day 72	No

^*a*^ Treatments are categorized based on their function, and information related to the administration dose is included when available. MIU, million international units. NA, not available.

Bacterial isolates from days 127 and 28 before the phage treatment were evaluated for phage susceptibility using spot test and liquid assays as described (10), with two phages showing an efficiency of plating (EOP) of 1 and a similar bacterial density control capacity in liquid culture (both related to the amplification strain). Based on previous favorable clinical outcomes (10), phage vB_Pae_10 (phage 10) was selected. Despite the potential heterogeneity of *P. aeruginosa* subpopulations, both isolates tested before and on day 0 showed equivalent phage susceptibility. By the end of December 2023, the Agencia Española de Medicamentos y Productos Sanitarios (AEMPS) approved the compassionate treatment. A 3 mL dose containing 10^10^ plaque-forming units (PFU), prepared as previously described (10), was nebulized daily for 10 days via eFlowrapid device (PARI Pharma GmbH) after physiotherapy and bronchodilators and was administered in combination with colistin (nebulized) and azithromycin (oral). The initial dose was medically supervised, and treatment continued at home without reported adverse effects. Fig. 1 summarizes the timeline of phage treatment and subsequent follow-up. After 10 days, a 1.2 log reduction in the bacterial burden was observed in morning-induced sputum. Concurrently, the %FEV1/FVC increased from 54.1% to 58%. In an attempt to eradicate the bacterium, an additional 10 days of nebulized treatment was administered, 4 days after finishing the initial round of treatment. During this second course, the residual volume (~1 mL) remaining in the reservoir of the nebulizer was applied locally in the upper respiratory tract via sinus rinses. There was no further reduction in the *P. aeruginosa* measured at day 21, and 3 days after phage treatment discontinuation (day 25), bacterial counts returned to the pre-treatment levels. However, the patient reported an overall improvement in her condition and the clearance of mucus in the upper respiratory tract following the nasal washing (not obtained with routine saline rinses). Concurrently, the %FEV1/FVC continued to increase during and shortly after the end of phage treatment on days 17 (59.9%) and 25 (62.6%).

**Fig 2:**
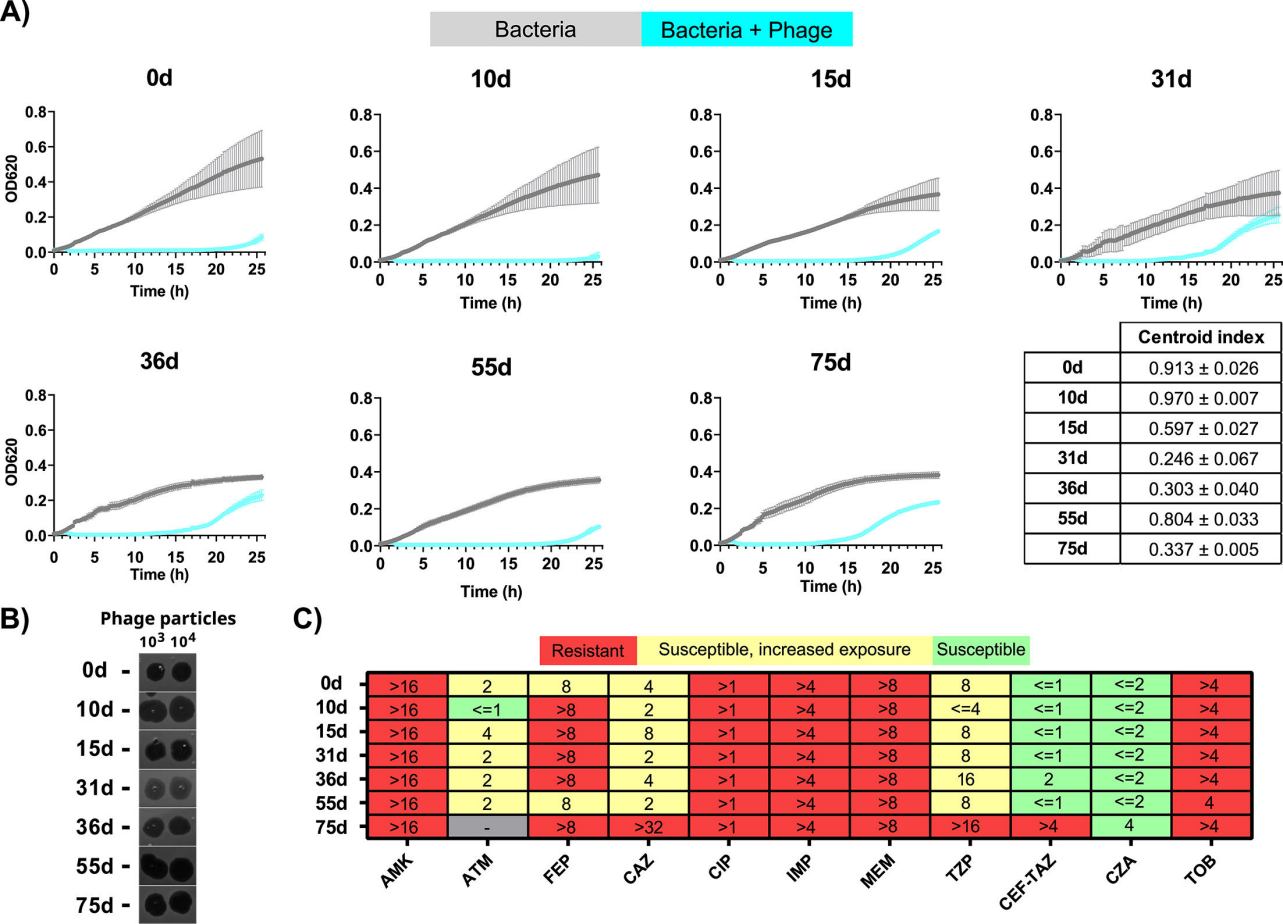
Susceptibility of clinical isolates before, during, and after phage treatment. (**A**) Phage 10 efficiency in liquid cultures. For each clinical isolate, 10^7^ CFUs were incubated in the absence and presence of phage 10 (multiplicity of infection [MOI] 10). Optical density (OD) at 620 nm was measured every 15 min and 30 s in three independent biological replicates, each containing two technical replicates (using the same bacterial culture). Mean values for the six replicates with standard deviation are represented in the growth curves. Mean values of two technical replicates were used to calculate the centroid index of each biological replicate independently. Mean centroid index values and standard error of the three biological replicates are shown in a table. (**B**) Phage 10 efficiency by spot test assays. For each clinical isolate, 10-fold phage dilutions were spotted in the bacterial lawn. Two representative spots containing different phage particles are shown. (**C**) Antibiograms. Minimum inhibitory concentration (μg/mL) determined in different clinical isolates by MicroScan broth microdilution panel NM57 (Beckman-Coulter) using EUCAST v13 breakpoints (2023). Color based on antibiogram result (green, susceptible; red, resistant; yellow, susceptible with increased exposure; gray, not assessed). AMK, amikacin; ATM, aztreonam; FEP, cefepime; CAZ, ceftazidime; CIP, ciprofloxacin; IPM, imipenem; MEM, meropenem; TZP, piperacillin-tazobactam; CEF-TAZ, ceftolozane-tazobactam; CZA, ceftazidime-avibactam; TOB, tobramycin.

To better understand the phage effect, bacterial isolates from different time points were analyzed. Notably, a slight change in phage susceptibility in liquid culture was observed during the second nebulization round (Fig. 2A). This was evidenced by the reduction of bacterial growth time in the presence of the phage *in vitro,* which correlated with a decrease in the centroid index, a metric that measures phage lytic efficiency (11), from 0.913 ± 0.026 on day 0 to 0.597 ± 0.027 on day 15. This phenotype was further pronounced in the following isolates, obtained at days 31 and 36 (centroid index of 0.246 ± 0.067 and 0.303 ± 0.040, respectively), which correlated with the rise of 1 log in bacterial load observed from day 21. However, the isolate on day 55 presented a centroid index of 0.804 ± 0.033, similar to the pre-treatment state, while, conversely, on day 75, it decreased to 0.337 ± 0.005. Spot assay revealed increased turbidity without EOP reduction on days 31 and 36, when the centroid index was lowest (Fig. 2B), but no turbidity on day 75 post-treatment, despite presenting a similar centroid index value. Antibiograms also revealed minor alterations in the antibiotic susceptibility of different isolates (Fig. 2C).

On day 34, acute rejection persisted (grade B2R), and the patient continued photopheresis and immunosuppressants. She remained stable until day 67, when cystic fibrosis transmembrane conductance regulator (CFTR) modulator treatment (elexacaftor/tezacaftor/ivacaftor) was initiated. Abdominal pain and nausea started on day 68, leading to hospital admission on day 72, with resolution of symptoms the same day after discontinuation of treatment.

During hospitalization, a transbronchial biopsy performed on day 75 showed no evidence of rejection. The *P. aeruginosa* isolated that day from a bronchoalveolar lavage showed an increased MDR phenotype, susceptible only to ceftazidime-avibactam, prompting its IV administration for 21 days. After the treatment, %FEV1/FVC increased to 67.9% on day 96, an improvement that persisted on day 180. Additionally, no *P. aeruginosa* was isolated from three sputum samples collected 123, 127, and 137 days. One year later, this patient’s sputum remained culture-negative for *P. aeruginosa* (data not shown).

No significant changes were observed in clinical indicators, such as blood cell counts, with lymphocyte concentration consistently low due to immunosuppressive treatment. C-reactive protein (CRP) levels decreased from 22 mg/L to the acceptable range (3–9 mg/L) after phage therapy, correlating with the initial reduction in *P. aeruginosa* load. Despite bacterial counts returning to pre-treatment levels, CRP remained stable, except on day 75 (20 mg/L) when the patient was hospitalized due to CFTR modulator adverse effects (Fig. 1B).

Phage detection in patient samples by nested PCR was performed, and immune response generation was evaluated using neutralization assays, as previously described (10). Phage screening in sputum and serum samples was positive in only 5/8 sputum samples collected during therapy (3 h, 7, 10, 15, and 17 days), with no detection after treatment completion (0/5 sputum samples up to day 53 and 0/3 serum samples up to day 66 post-treatment). As expected, given the immunosuppressed state of the patient, no serum or sputum samples showed phage-neutralizing activity, even 66 days post-treatment initiation.

## DISCUSSION

This case report presents a successful treatment with favorable clinical and microbiological outcomes following phage therapy in combination with antibiotics. Treatment was deemed safe, as no adverse effects were observed. Clinical improvement was noted shortly after phage therapy initiation, evidenced by a reduction in mucus and an increase in the %FEV1/FVC, accompanied by a decrease in the bacterial load, as previously reported in CF patients receiving similar phage treatments against *P. aeruginosa* (6). This improvement was sustained despite the return to pre-treatment bacterial levels on day 25. Positive outcomes despite the non-eradication of bacteria have also been documented (10, 12) and have been proposed to be a consequence of bacterial trade-offs (reduced virulence or antibiotic resistance) resulting from the phage treatment (6, 13).

Here, phage intervention, in combination with antibiotics and immunosuppressive treatment, contributed to the modulation of the *P. aeruginosa* population and its phenotype. Although sequencing could provide deeper insights into the dynamics between subpopulations, evidence of this modulation was observed through slight variations in phage and antibiotic susceptibility among bacterial isolates. The heterogeneity of phage susceptibility may result from hypermutator variants selected by phage exposure, as previously observed (12). Additionally, minor changes in antibiogram profiles could suggest the presence of diverse subpopulations. Notably, discrepancies between the phage susceptibility methods on day 75 isolate highlight the need for further research to determine the most reliable approach. Changes in *P. aeruginosa* population could be associated with a sustained lung capacity improvement and a reduction in the patient’s inflammatory state, having a potential impact on the disappearance of acute rejection on day 96 and the absence of bacteria in sputum after ceftazidime-avibactam administration. Both conditions (no rejection and no isolation of *P. aeruginosa* in sputum) remained stable at least 1 year after the treatment. Following therapy, phage neutralization was not observed, presumably, as a consequence of the immunosuppressive patient status (14, 15). These findings support the hypothesis that the immune response after nebulized therapy is influenced not only by the phage but also by patient factors, such as immune status. Indeed, phage 10 has been shown to trigger neutralizing antibody production after 10 days in an immunocompetent patient (10).

Overall, phage treatment in combination with antibiotics led to clinical improvement and an increase in %FEV1/FVC, which could have contributed to the subsequent clearance of bacteria from sputum and resolution of the acute rejection status. The extent to which the phage contributed to this outcome is difficult to determine, given that it was administered concurrently with antibiotics against *P. aeruginosa* and immunosuppressants to prevent rejection. However, phage nebulization was a turning point in the clinical condition of the patient, which had not improved for months with the conventional treatments alone. Although nebulized phage therapy has been shown to reduce bacterial burden in CF lung infections, thereby improving lung function and patient quality of life, prolonged exposure to high-titer phages may lead to the selection of variants with reduced lytic activity, potentially impacting clinical outcomes. Controlled clinical trials are required to optimize dosage, duration, and efficacy of phage therapy as well as the implication of phage resistance and immune response to clinical outcomes.
